# Maternal Vitamin D Deficiency is Associated With Sex‐Dependent Alterations in Neurodevelopment‐Related Gene Expression, Particularly in the Hypothalamus

**DOI:** 10.1002/mnfr.70633

**Published:** 2026-10-25

**Authors:** Lidia Saidi, Emmanuel Snacel‐Fazy, Eva Seipelt, Nicole Haroun, Julien Astier, Charlène Couturier, Mohamed El Soltani, Eric Sérée, Flavie Sicard, Habib Hammou, Michel Puceat, Jean‐François Landrier, Lourdes Mounien

**Affiliations:** ^1^ Aix‐Marseille Univ INSERM INRAE C2VN Marseille France; ^2^ Laboratory of Physiology of Nutrition and Food, Safety Department of Biology Faculty of Natural and Life Sciences Oran 1 University Oran Algeria; ^3^ PhenoMARS Aix‐Marseille Technology Platform Marseille France

**Keywords:** brain development, hippocampus, hypothalamus, maternal vitamin D deficiency, melanocortin pathway, vitamin D

## Abstract

Vitamin D (VD) is essential for calcium homeostasis and bone metabolism but also for brain development and function. However, few data are available on the impact of maternal VD deficiency (VDD) on sex‐specific brain development, particularly in specific structures such as hypothalamus. This study investigated the impact of maternal VDD on early brain development, neuronal and glial differentiation, and hypothalamic gene expression in mice. Maternal VDD was associated with increased mRNA expression of markers related to neuronal differentiation and synaptogenesis in both sexes, while mRNA expression of astrocyte‐related markers remained largely unchanged. Male offspring exhibited broader transcriptomic alterations, including dysregulation of melanocortin‐pathway and synaptic signaling genes, in the hypothalamus of early postnatal and adult mice. Maternal VDD was associated with sex‐dependent changes in gene expression relevant to early brain development, with altered mRNA expression of markers involved in neuronal differentiation and synaptogenesis in both sexes. Male offspring exhibited transcriptomic changes, including altered expression of melanocortin‐pathway and synaptic signaling genes, further suggesting greater susceptibility of hypothalamic gene expression to maternal VDD. These findings indicate that maternal VDD may influence neurodevelopmental gene expression during early life, potentially contributing to later alterations in synaptic function, metabolism, and behavior.

## Graphical abstract

Maternal vitamin D deficiency (VDD) affects early brain development in a sex‐dependent manner. VDD accelerates neuronal differentiation and synaptogenesis. Male offspring exhibit broader transcriptomic alterations, including dysregulation of melanocortin‐pathway and synaptic signaling genes, indicating greater vulnerability of hypothalamic circuits. These findings suggest that maternal VDD reprograms neurodevelopmental pathways, with potential long‐term consequences for synaptic function, metabolism, and behavior.

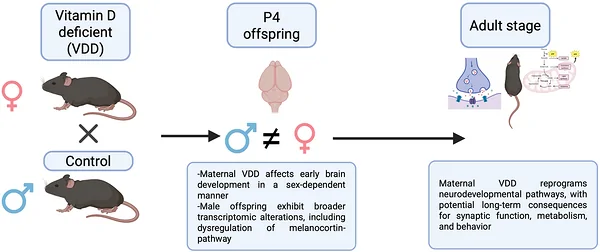

## 1 Introduction

Vitamin D (VD) is widely recognized as an essential hormone for calcium homeostasis and bone metabolism [1, 2]. However, its role has expanded significantly beyond skeletal functions to encompass numerous non‐skeletal activities, including cell differentiation, proliferation, and apoptosis, immune regulation, and more recently, brain development and function [3]. The involvement of VD in brain development and function is supported by the early presence of the VD receptor (VDR) during embryonic development, appearing as early as Day 12 of gestation in various developing brain regions, including the mesencephalon [4]. VDR expression continues throughout life, being detected in the central nervous system (CNS) of neonatal and adult brains, as well as in various brain cell types, including neurons, astrocytes, and microglia [5, 6, 7]. Furthermore, the presence of 1‐α hydroxylase (CYP27B1), the enzyme responsible for synthesizing the active form of VD, has been documented in brain tissue, indicating local VD synthesis capacity within the CNS [5].

VD has a crucial role in regulating neuronal stem cell proliferation and differentiation, with studies demonstrating that VD deficiency promotes excessive proliferation while reducing apoptosis in multiple brain regions [8, 9]. Additionally, VD enhances the expression of several neurotrophic factors, including nerve growth factor (NGF), glial cell‐derived neurotrophic factor (GDNF), and brain‐derived neurotrophic factor (BDNF) [8, 9, 10]. In addition, VD exerts modulatory effects on neurotransmitter systems. Particularly, it has been highlighted that VD influences the release of glutamate and gamma‐aminobutyric acid (GABA) [11, 12], and regulates serotonin and dopamine (DA) release by modulating the expression of their rate‐limiting enzymes, tryptophan hydroxylase 2 (TPH2) and tyrosine hydroxylase (TH), respectively [13, 14].

In the context of the high prevalence of VD deficiency (VDD) among pregnant women and the established involvement of VD in various developmental disorders, numerous studies have investigated the impact of maternal VDD on offspring neurodevelopment. For instance, maternal VDD has been associated with alterations in the expression of genes involved in neurodevelopment. In rats, maternal VDD resulted in changes in the expression of numerous genes involved in cell cycle regulation [15]. Similarly, VD restriction during fetal growth was associated with alterations in genes essential for cytoskeleton maintenance, such as MAP2, and neurotransmission, including GABA‐Aα4 [16]. In a Sprague‐Dawley rat maternal VDD model, it has been observed a decrease of the expression of Nurr1 (nuclear receptor‐related 1) and p57Kip2 (p57 kinase inhibitory protein 2), both essential factors for the development of the dopaminergic phenotype [17]. Beyond molecular changes, maternal VDD has also been associated with morphological alterations in the brains of offspring. For example, offspring of VDD rats exhibited larger brains with enlarged lateral ventricles, whereas progeny of VDD female mice displayed smaller brains and lateral ventricles [3]. These alterations in brain chemistry and morphology were associated with behavioral changes. Particularly, offspring of VDD female mice and rats consistently demonstrated signs of spontaneous locomotor hyperactivity [18, 19, 20]. It has been also shown that maternal VDD programs reproductive dysfunction in adult female offspring through adverse effects on hypothalamic function [21]. Interestingly, it has been observed that the expression of hypothalamic melanocortin pathways genes including Neuropeptide Y (NPY) and Agouti‐related peptide (AgRP) is different in maternal VDD group compared to control animals [18]. These alterations in the expression of melanocortin pathway genes were associated with changes in food intake [18].

Taken together, these observations suggest that VD exerts an important effect in the development of the CNS. However, few data are available on the sex‐specific development of brain structures as hypothalamus associated with the adverse effects of maternal VDD and particularly at the early stage of life. Therefore, the aim of the present study was to determine the impact of maternal VDD on the offspring brain development and signaling pathways during the early stages of life and the long‐term effect of this effect on brain function with a particular focus on the hypothalamus.

## 2 Materials and Methods

### 2.1 Experimental Animals

All experimental procedures received approval from both the Aix‐Marseille University Ethics Committee and the French Ministry of Research (APAFIS#1300‐2015072112279135). C57BL/6JRJ mice of both sexes were procured from Janvier Labs (Le Genest‐Saint‐Isle, France) and housed under standard laboratory conditions with unrestricted access to standard rodent chow (A04, Safe‐diets, Augy, France) and water during a 1‐week acclimatization period. Environmental conditions were maintained at 22°C with a 12:12 h light‐dark cycle and 20% humidity. Following acclimatization, female mice (*n* = 15 per group) were randomly allocated to one of two dietary treatment groups: a control diet containing vitamin D3 (AIN‐93G, 1.0 IU g^−1^) or a vitamin D‐depleted diet (AIN‐93G, 0.0 IU g^−1^). These dietary treatments were administered for 8 weeks prior to mating with male mice, following established protocols [18, 22, 23]. Post‐delivery, all dams received the standard control diet (AIN‐93G) throughout the lactation period until offspring weaning. Litter sizes were standardized to six pups per dam to ensure uniform nutritional conditions. The P4 offspring used in this study were derived from multiple independent litters (7 control litters and 4 VDD litters). More than one pup from a given litter could be used for a specific analysis.

Postnatal brains of pups were collected 4 days (P4) after birth. After decapitation, the brains (11 male and 15 female controls; seven male and six female VDD) were collected and stored at −80°C for subsequent analyses.

At 6 weeks of age, male and female offspring (*n* = 6–8 per group) were randomly distributed into standard diet (NC, AIN‐93 M Maintenance Purified Diet) obtained from Safe‐diets (Augy, France). These post‐weaning dietary treatments continued for 8 weeks. Following overnight fasting, animals were euthanized via cervical dislocation, and hypothalamus were collected and stored at −80°C for subsequent analyses.

### 2.2 Sexing of Dams by PCR

The genetic sex of mice is determined using the DirectPCR Lysis Reagent kit (Tail). DNA extraction from skin is performed by cell lysis in a suitable reagent in the presence of 0.4 mg/mL of Proteinase K on a Thermomixer at 55°C overnight, followed by inactivation at 85°C for 45 min. PCR is then performed on the lysate to amplify the Rbm31Y and Rbm31X genes. PCR is performed on 1 µL of lysate for 35 cycles (each cycle consists of 30 s denaturation at 94°C, followed by 30 s hybridization at 60°C and 30 s elongation at 72°C). Visualization is performed on a 2% TAE 1X agarose gel, and migration is performed for 10 min at 50 V, then between 60 min and 80 min at 100 V.

Females possess only copies of Rbm31X, visible as a single band at 269 base pairs, while males possess both versions of the gene, amplified at different sites, resulting in two amplicons of different sizes, revealing two bands at 269 and 353 base pairs. The primer sequences utilized in this investigation are detailed in the Supporting Information (Table S1).

### 2.3 RNA Extraction and Real Time PCR

RNA isolation was performed from total brain at P4 and adult hypothalamus samples using TRIzol reagent (Thermo Fischer Scientific, Les Ulis, France). cDNA synthesis was conducted using 1 µg of hypothalamic total RNA as template, employing random primers in combination with Moloney murine leukemia virus reverse transcriptase (Thermo Fischer Scientific, Les Ulis, France).

Quantitative real‐time PCR amplifications were carried out on the Mx3005P Real‐Time PCR System (Stratagene, La Jolla, USA) according to established methodologies [18, 22, 23, 24]. Gene expression levels were assessed in duplicate for each experimental condition, with 18S rRNA serving as the internal reference gene for normalization using the comparative cycle threshold (CT) approach. All primer sequences utilized in this investigation are detailed in the supplementary materials (Table S1).

### 2.4 RNA Sequencing

Total RNA from three mice per group were used to prepare an RNA‐seq library using the Illumina TruSeq Stranded mRNA kit. On the Illumina NextSeq 500 sequencer, libraries were sequenced paired‐end. Sickle was used to eliminate reads having a phred score of less than 20 and a length of less than 25 bp (v1.33). MultiQC was used to assess the quality of the trim reads (v1.0). Trim readings were aligned with the STAR aligner (v2.7.0d) with the “out Filter Mismatch Nover Lmax” and “out Filter‐Multimap Nmax” options set to 0.08 and 1, respectively. Cufflinks (v2.2.1) was used to find transcripts, with the “library‐type” option set to fr‐firstrand and a GTF file obtained from GENCODE (“Comprehensive gene annotation”, vM1) serving as the genomic annotation. Cuffmerge was used to integrate the GTF files created by Cufflinks for each sample.

Unknown transcripts (class code “u”) were identified using the “class code” supplied by Cuffmerge to each transcript. For further analysis, only de novo transcripts with counts greater than zero in at least one RNA‐seq sample were preserved. These newly created transcripts were joined with the GENCODE GTF file to create the final genomic annotation, which was then quantified using FeatureCounts (v1.6.1). DESEQ2 was used to compare gene expression patterns between conditions. Reads from Watson and Crick strands were picked using SAMtools (v1.9) and sent into the RseQC program suite's bam2wig.py script to build bigwig files (v2.6.4). The IGV genome browser was used to visualize RNA‐seq profiles.

### 2.5 Ingenuity Pathway Analysis (IPA)

Differential expression data were filtered using the following parameters: an adjusted *p*‐value (*padj*) < 0.01, with *p*‐values corrected for multiple testing using the Benjamini–Hochberg method, and an absolute log2 fold‐change threshold of (|log2FC|) > 1. The resulting gene lists were submitted to Ingenuity Pathway Analysis software (IPA, Qiagen Redwood City, Inc., Redwood City, CA, USA) to identify neuronal and canonical pathways associated with the transcriptomic changes observed following maternal VDD. Pathways were manually curated to retain only those related to neuronal and canonical in our study.

### 2.6 Statistical Analyses and the Use of Artificial Intelligence

All data are expressed as mean ± SEM. Statistical analysis was performed by using GraphPad Prism (version 10, GraphPad software LLC, San Diego, CA, USA). The statistical tests used (unpaired Student's *t*‐test or Mann‐Whitney test) are indicated in the legend of each figure. Data distribution was assessed using a normality test (Shapiro‐Wilk). For the transcriptomic data, a *padj* < 0.01 was used to define differentially expressed genes, whereas *p* < 0.05 was considered statistically significant for the other statistical analyses.

Multiple qPCR comparisons were performed without correction for multiple testing; therefore, a potential increase in error cannot be excluded. Because statistical analyses were performed at the individual offspring level and litter was not included as a random effect, a potential contribution of litter‐specific effects cannot be completely excluded.

Artificial intelligence was used for text editing.

## 3 Results

### 3.1 Maternal Vitamin D Deficiency Modulates the mRNA Expression of Genes Involved in General Brain Development in Early Postnatal Mice

To investigate whether maternal vitamin D deficiency (VDD) affects brain development in P4‐aged male and female offspring, we analyzed the expression of genes associated with general brain maturation. The levels of Neuronal Nuclear Protein (*NeuN*), Neurogenic Differentiation 1 (*NeuroD1*), and Tyrosine Hydroxylase (*TH*) gene expression were significantly upregulated in both VDD males and females compared to control mice (Figure 1A,C,D). Additionally, the expression of Brain‐Derived Neurotrophic Factor (*BDNF*) was significantly increased in VDD males only (Figure 1E). In contrast, no significant change was observed in the expression of Glial Fibrillary Acidic Protein (*GFAP*) between the VDD and control groups (Figure 1B).

**FIGURE 1:**
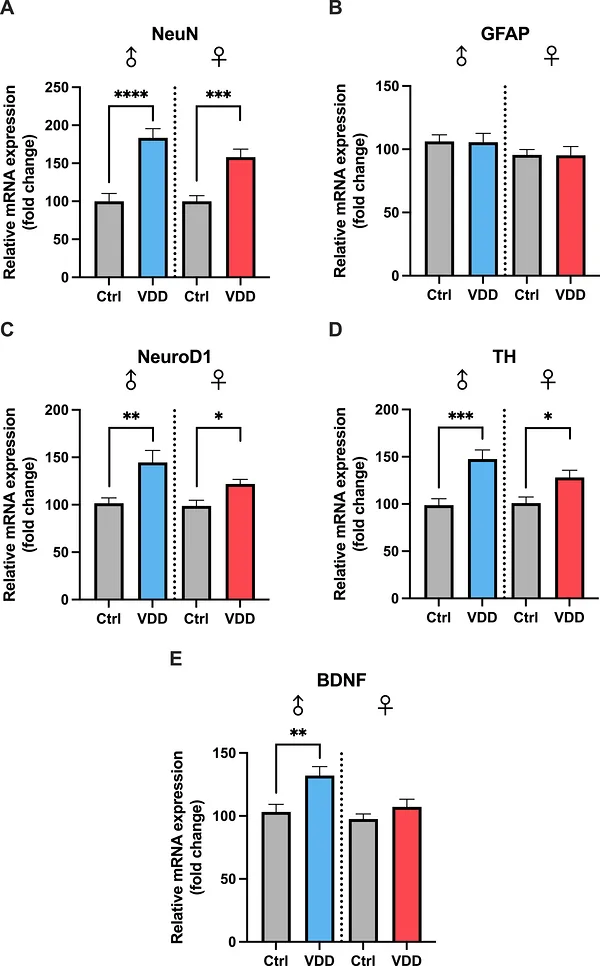
Maternal vitamin D deficiency altered general brain development gene expression at P4. Effect of maternal VDD on the levels of Neuronal Nuclear Protein (*NeuN*) (A), Glial Fibrillary Acidic Protein (*GFAP*) (B), Neurogenic Differentiation 1 (*NeuroD1*) (C), Tyrosine Hydroxylase (*TH*) (D) and Brain‐Derived Neurotrophic Factor (*BDNF*) (E) in male and female offspring at P4. Ribosomal protein 18S rRNA was used as the endogenous control. Data were compared with Mann‐Whitney (**p < 0.01, ***p < 0.001, ***p < 0.001, ****p < 0.001) and are presented as mean ± standard error of the mean (SEM). *N* = 10 ctrl male; 6 VDD male; 13 ctrl female, 5 VDD female. Ctrl: control; VDD: vitamin D deficiency. Ctrl: control; VDD: vitamin D deficiency.

### 3.2 Maternal Vitamin D Deficiency Alters Synaptogenesis‐Related Gene mRNA Expression in Early Postnatal Mice

We next examined the expression of key synaptogenesis‐related genes, including Postsynaptic Density Protein 95 (*PSD95*), Synapsin‐1 (*Syn‐1*), and Synaptophysin (*Syp*), in the brains of VDD and control animals at P4. *PSD95* expression was upregulated in both VDD male and female offspring compared to controls, whereas *Syp* expression remained unchanged between groups (Figure 2A,B). A significant increase in *Syn‐1* expression was also observed in VDD males but not in females compared to the control group (Figure 2C).

**FIGURE 2:**
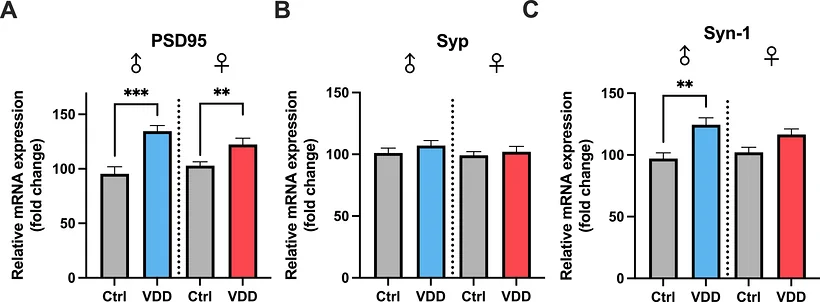
Maternal vitamin D deficiency altered synaptogenesis‐related gene expression at P4. Effect of maternal VDD on the levels of Postsynaptic Density Protein 95 (*PSD95*) (A), Synaptophysin (*Syp*) (B) and Synapsin‐1 (*Syn‐1*) (C) in male and female offspring at P4. Ribosomal protein 18S rRNA was used as the endogenous control. Data were compared with Mann‐Whitney (**p < 0.01, ***p < 0.001) and are presented as mean ± standard error of the mean (SEM). *N* = 10 ctrl male; 6 VDD male; 13 ctrl female, 5 VDD female. Ctrl: control; VDD: vitamin D deficiency.

### 3.3 Region‐Specific Gene Expression Change Is Associated With Maternal Vitamin D Deficiency in Early Postnatal Mice

In a first analysis, we evaluated the impact of maternal VDD on the expression of Single‐minded 1 (*Sim1*) and Steroidogenic Factor 1 (*SF1*), both involved in hypothalamus development. A significant upregulation of *Sim1* expression was observed exclusively in VDD males at P4 compared to controls, while *SF1* levels remained unchanged between groups (Figure 3A,B).

**FIGURE 3:**
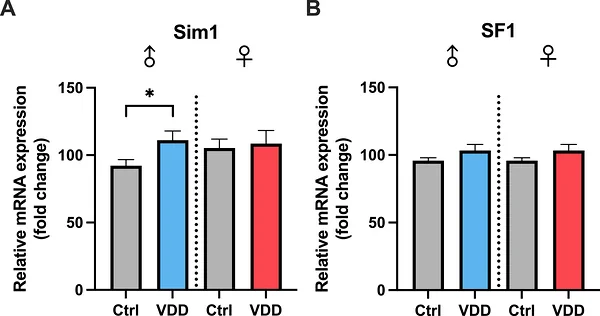
Maternal vitamin D deficiency altered hypothalamus development gene expression in male offsprings at P4. Effect of maternal VDD on the levels of Single‐minded 1 (*Sim1*) (A) and Steroidogenic Factor 1 (*SF1*) (B) in male and female offspring at P4. Ribosomal protein 18S rRNA was used as the endogenous control. Data were compared with Mann‐Whitney (*p < 0.05) and are presented as mean ± standard error of the mean (SEM). *N* = 10 ctrl male; 6 VDD male; 13 ctrl female, 5 VDD female. Ctrl: control; VDD: vitamin D deficiency.

We also assessed the expression of genes crucial for hippocampal development, including Disks Large Homolog 3 (*DLG3*), Dopamine Receptor D3 (*DRD3*), and LY6/PLAUR Domain Containing 1 (*LYPD1*). Among these, only *DRD3* showed a significant increase in expression in both VDD males and females compared to controls (Figure S1).

### 3.4 Maternal Vitamin D Deficiency Is Associated With Sex‐Specific Alterations in the Expression of Melanocortin Pathway Genes in Early Postnatal Mice

As previously reported, body weight was affected in both male and female offspring born to VDD dams [18] (Figure S2). This impairment may be related to an impact of VDD on the melanocortin pathway.

Then, to further investigate this hypothesis, we assessed the mRNA expression levels of genes associated with the melanocortin pathway: *NPY* (neuropeptide Y), *AgRP* (agouti‐related peptide), and *POMC* (pro‐opiomelanocortin) in male and female offsprings at P4. Male pups, but not females, showed a significant increase in the expression of *NPY*, *AgRP*, and *POMC* (Figure 4).

**FIGURE 4:**
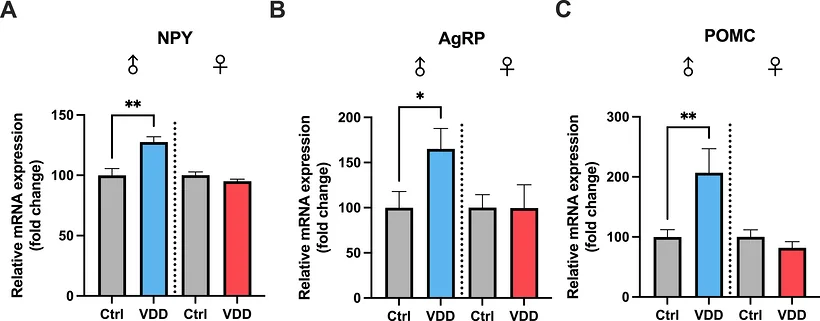
Maternal vitamin D deficiency led to sex‐specific modulation of melanocortin pathway genes at P4. Effect of maternal VDD on the expression of Neuropeptide Y (*NPY*) (A), Agouti‐related peptide (*AgRP*) (B) and pro‐opiomelanocortin (*POMC*) (C) in male and female offspring at P4. Ribosomal protein 18S rRNA was used as the endogenous control. Data were compared with Mann‐Whitney (*p < 0.05, **p < 0.01) and are presented as mean ± standard error of the mean (SEM). *N* = 10 ctrl male; 6 VDD male; 13 ctrl female, 5 VDD female. Ctrl: control; VDD: vitamin D deficiency.

### 3.5 Maternal Vitamin D Deficiency Is Associated With Sex‐Specific Modulation of Hypothalamic Gene Expression in Adult Mice

RNA sequencing analysis was conducted to assess the impact of maternal VDD on gene expression profiles in the hypothalamus of adult male and female mice. Differentially expressed genes (DEGs) were defined using a *padj* < 0.01 and an absolute log2 fold‐change (|log2FC|) > 1. In adult male offspring from VDD dams, a total of 701 genes were differentially expressed compared to controls, including 226 upregulated and 475 downregulated transcripts (Figure 5A). In contrast, female offspring exhibited a smaller transcriptomic response, with 137 differentially expressed genes: 48 upregulated and 89 downregulated (Figure 5B).

**FIGURE 5:**
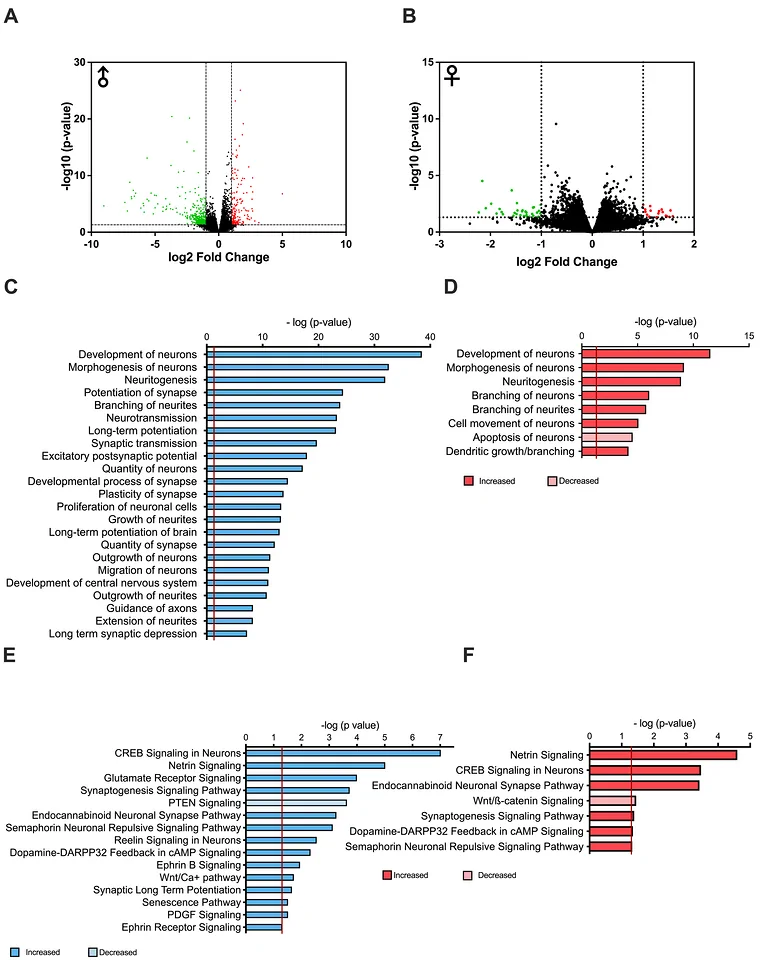
Maternal vitamin D deficiency was associated with sex‐specific alterations in hypothalamic gene expression in adult mice. Volcano plot of differentially expressed hypothalamic genes between control versus VDD adult male (A) and female (B) offspring. Differentially expressed genes were identified using an adjusted *p*‐value (padj) < 0.01 and an absolute log2 fold‐change threshold of (|log2FC|) > 1. IPA analyses of neuronal and canonical pathways associated with the differentially expressed genes in male (C,E) and female (D,F) offspring. Bar charts represent the most significant pathways identified by IPA; values are presented as ‐log(*p*‐value).

Using IPA analysis, we identified 23 neuronal (Figure 5C) and 15 canonical pathways (Figure 5E) that were differentially expressed between control and VDD male offspring. In females, 8 neuronal (Figure 5D) and 7 canonical pathways (Figure 5F) were differentially expressed between the control and VDD groups. Notably, 5 neuronal and 5 canonical pathways were found to be common between males and females.

## 4 Discussion

In our study, we observed that maternal VDD significantly altered the mRNA expression of genes associated with early brain development and hypothalamic function in a sex‐dependent manner. Our results revealed that offspring born to VDD dams exhibit altered mRNA expression of genes involved in general neurodevelopment, synaptogenesis, and region‐specific brain maturation, with more pronounced transcriptomic alterations in male mice.

In this study, we investigated how maternal VDD influences early postnatal brain development in mice at postnatal Day 4 (P4), focusing on key markers of neuronal and glial differentiation. We observed a significant upregulation of *NeuN* mRNA expression in the brains of offspring from VDD dams, whereas no significant difference was found in *GFAP* expression. The increase in NeuN mRNA level, a marker of mature postmitotic neurons, may reflect changes in the expression of genes associated with neuronal differentiation or maturation in the developing brain. Mechanistically, VD has been shown to influence several neurodevelopmental pathways. The active form of VD, 1,25‐dihydroxyvitamin D_3_, binds to the VDR, which is expressed in the embryonic and early postnatal brain, particularly in regions such as the cortex, hippocampus, and cerebellum. VDR regulates gene transcription involved in calcium homeostasis, neurotrophin signaling and pathways such as Wnt and Sonic Hedgehog, all of which are critical for neuronal proliferation, migration, and differentiation [25]. A deficiency in maternal VD could therefore be associated with altered activity of these pathways and changes in the timing or extent of neuronal development. In accordance with this hypothesis, it has been shown that maternal VDD in rats resulted in a longer and larger brain, a longer and thinner cortex and enlarged lateral ventricles, which persist into adulthood [3, 8, 15, 16]. In contrast, we observed no significant difference in *GFAP* expression, coding for a canonical marker of differentiated astrocytes. Astrocyte development typically occurs slightly later than neuronal differentiation, and while some astrocytes are present at P4, the lack of change in *GFAP* mRNA expression may suggest that astrocyte‐related gene expression is not modified by prenatal VD levels at this stage. Taken together, our findings suggest a selective and early effect of maternal VDD on the expression of genes involved in neuronal lineage commitment or maturation, without concomitant changes in astrocyte‐related gene expression at this stage. These results are consistent with previous studies reporting alterations in cortical development and neuronal density, as well as behavioral changes, in rodents exposed to prenatal VDD [16, 17]. In addition, we observed a significant upregulation of *NeuroD1* and *TH* mRNA expression in both male and female P4 offspring exposed to maternal VDD, suggesting altered expression of genes involved in neuronal differentiation. The increase in *BDNF* mRNA expression observed exclusively in males may indicate a sex‐specific transcriptional response to maternal VD deprivation, potentially relevant to neurotrophic signaling. This hypothesis is consistent with earlier evidence from animal models showing that prenatal VDD was associated with changes in the expression of neurotrophin‐related genes, such as *BDNF* and *Tgf‐β1* (transforming growth factor‐β1) and dopaminergic markers such as *TH* in the fetal brain of male BALB/c mice [26].

In addition, maternal VDD was associated with increased PSD95 and Syn‐1 mRNA expression in both sexes, suggesting potential changes in the expression of genes involved in synaptic formation or remodeling. This is consistent with previous studies showing that VD can regulate synaptic plasticity and neurotransmission through calcium‐dependent pathways [27, 28]. Proteomic studies indicated that that VDD during development was associated with altered expression of proteins involved in synaptic plasticity and neurotransmission [29]. The lack of change in *Syp* mRNA expression suggests that not all genes involved in synaptic components are equally sensitive to maternal VDD. These differential effects could reflect compensatory mechanisms to maintain synaptic integrity under suboptimal developmental conditions.

Our analysis also revealed distinct region‐specific changes in gene expression associated with maternal VDD. The mRNA upregulation of *Sim1* in the hypothalamus of VDD males, with no change in *SF1* expression, suggests that VD deficiency may selectively change the expression of genes associated with specific neuronal subpopulations involved in hypothalamic development. In the hippocampus, the increased *DRD3* mRNA expression in both sexes further supports the notion that dopaminergic‐related gene expression is particularly sensitive to prenatal VDD. Such changes may have long‐term implications for emotional regulation and cognitive function, consistent with previous studies exhibiting a link between maternal VD status and neuropsychiatric outcomes [3, 29, 30].

A salient result is the sex‐specific modulation of hypothalamic gene expression, particularly within the melanocortin pathway. Male offspring, but not females, exhibited a significant mRNA upregulation of *NPY*, *AgRP*, and *POMC*, suggesting an early alteration in the expression of genes belonging to hypothalamic circuits controlling energy balance and feeding behavior. This pattern suggests that male hypothalamic gene expression may be more susceptible to maternal VDD, potentially predisposing to later metabolic and neuroendocrine alterations. In line with this hypothesis, body weight and energy expenditure were differentially changed in juvenile male and female offspring born to VDD dams [18]. Moreover, our transcriptomic analysis in adults revealed a broader differential gene expression in males compared with females, reinforcing the idea of long‐term sex‐dependent transcriptional programming associated with maternal VDD. Interestingly, in adulthood, a high‐fat diet combined with maternal VDD was associated with further alterations in adiposity in male offspring but not in females [18]. This phenotype appears to be associated with a persistent dysregulation of the melanocortin pathway that may originate early in life.

These findings suggest that the increased sensitivity of the male hypothalamus to VD deficiency may be linked to the critical window of brain masculinization. During this period, testosterone, partly through its aromatization into estradiol, plays a central role in shaping hypothalamic differentiation [31, 32]. In parallel, VD signaling via the VDR is known to regulate several key neurodevelopmental processes. A deficiency in VD during this stage may therefore interfere with these coordinated pathways and preferentially impact male brain development [3]. Although the underlying mechanisms remain to be fully elucidated, emerging evidence points toward a potential interaction between VDR and sex steroid receptors, possibly mediated by shared nuclear co‐regulators, which could modulate transcriptional activity [33]. The sex‐specific differences in developmental timing may further contribute to this increased male vulnerability [34].

To further explore these sex‐dependent molecular differences, we performed RNA‐sequencing and subsequent IPA of adult hypothalami. Interestingly, the genomic studies demonstrate that VD regulates thousands of genes in a cell‐type and species‐specific manner, through the action of the VDR as a transcription factor [35]. This observation underlines the fact that maternal VDD may contribute to aberrant programming of gene expression in brain regions critical for metabolism and behavior. In agreement, we identified 23 neuronal and 15 canonical pathways that were differentially expressed between control and VDD male offspring, whereas 8 neuronal and 7 canonical pathways were altered in females. Interestingly, 5 neuronal and 5 canonical pathways were found to be common between males and females, suggesting that although both sexes are affected by maternal VDD, males display a broader and more complex transcriptomic response. This sex bias in gene regulation reinforces the hypothesis that male hypothalamic circuits are more vulnerable to prenatal VD deficiency.

We identified several conserved neuronal pathways enriched among differentially expressed genes in both male and female offspring, including those related to neuron development, neuronal morphogenesis, neuritogenesis and neurite growth and branching. These findings suggest that maternal VDD alters the expression of genes involved in fundamental processes of neuronal maturation and circuit formation early in life. This is consistent with our targeted gene expression analyses, which showed increased mRNA expression of genes associated with neuronal development, including *NeuN*, *NeuroD1*, and *TH*, in both male and female P4 offspring, whereas *BDNF* was increased in males only. Together, these results indicate that maternal VDD is associated with early dysregulation of the expression of genes involved in neuronal differentiation and maturation, which may have lasting consequences for brain function.

One of the most interesting observations in male offspring is the selective differentially expression of genes belonging to pathways related to synaptic signaling and plasticity, potentiation of synapses, long‑term potentiation (LTP), synaptic transmission, excitatory postsynaptic potential (EPSP), plasticity of synapses, and long‑term synaptic depression (LTD). The involvement of these pathways suggests that maternal VDD may alter the expression of genes involved not only in neuronal development, but also in those regulating synaptic strength and efficacy. Indeed, previous work in animal models has shown that VDD was associated with attenuated LTP in the hippocampus. For instance, rats deprived of VD showed only moderate EPSP potentiation after tetanic stimulation compared with control rats [36]. Moreover, VD has been shown to preserve synaptic transmission and plasticity in aging or neurodegenerative contexts. In fact, higher VD intake prevented age‑related synaptic decline and enhanced hippocampal synaptic function [37]. In our study, the increased mRNA expression of genes associated with neuronal maturation (*NeuN, NeuroD1*, and *TH*) and synaptogenesis (*PSD95, Synapsin‑1*) in male offspring may reflect premature or altered synaptic maturation, potentially limiting the capacity for dynamic plastic changes such as LTP and LTD. The male‐biased transcriptomic response found via RNA‐seq further supports this hypothesis, reflecting a wider reprogramming of the expression of genes involved in synaptic signaling pathways in males than in females. If synaptic potentiation and depression mechanisms are altered early in life, this could compromise hypothalamic circuits’ ability to respond adaptively to metabolic or environmental cues. Consequently, the observed alterations in mRNA expression of melanocortin‐pathway genes (*NPY, AgRP*, and *POMC*) in male offspring may represent an early downstream consequence of altered expression of genes involved in synaptic plasticity within hypothalamic feeding and energy balance circuits. Future studies should directly assess electrophysiological read‑outs (e.g., EPSP amplitude, LTP induction, and LTD magnitude) in hypothalamic slices of VDD male offspring and determine whether supplementation of VD or modulators of synaptic plasticity postnatally can rescue both synaptic and metabolic outcomes.

Our pathway analysis revealed that maternal VDD modulates the expression of genes involved in several canonical signaling cascades known to regulate neuronal communication and structural remodeling. Among these, several signaling pathways including ephrin receptor or semaphorin signaling were found to be common between male and female offspring. For instance, these pathways play essential roles in axon guidance, synapse formation, and the refinement of neural circuits during early development [38, 39]. However, males born to VDD dams displayed a broader and more complex transcriptomic alteration, with numerous additional canonical pathways affected such as glutamate receptor, Wnt/Ca+ and PTEN (Phosphatase and TENsin homolog) pathways. This male‐specific enrichment of signaling alterations suggests that male brains are more responsive or more vulnerable to maternal VDD at the molecular level.

In conclusion, maternal VDD was associated with pronounced, sex‐dependent changes in gene expression relevant to early brain development. Maternal VDD was associated with increased mRNA expression of markers related to neuronal differentiation and synaptogenesis (NeuN, NeuroD1, TH, PSD95, and Syn‐1), while mRNA expression of astrocyte‐related markers remained largely unchanged. Male offspring exhibited broader transcriptomic alterations, including changes in the expression of melanocortin‐pathway and synaptic signaling genes, indicating greater molecular vulnerability of hypothalamic circuit‐related gene expression. These findings suggest that maternal VDD alters the expression of genes involved in key neurodevelopmental pathways, with potential long‐term consequences for synaptic function, metabolism, and behavior. However, as these conclusions are based primarily on mRNA expression profiles, further studies incorporating protein‐level, morphological, and functional analyses will be required to confirm these biological outcomes.

## Author Contributions

L. Mounien, J.F. Landrier, and M. Puceat designed research. L. Mounien, H. Hammou, and L. Saidi analyzed data. L. Mounien, J.F. Landrier, L. Saidi, J. Astier, E. Snacel‐Fazy, C. Couturier, M. El Soltani, E. Seipelt, N. Haroun, E. Serée, and F. Sicard, performed research and help in the paper writing. L. Mounien and L. Saidi wrote the paper .

## Funding

The authors have nothing to report.

## Conflicts of Interest

The authors declare no conflicts of interest.

## Supporting information




**Supporting File**: mnfr70633‐sup‐0001‐SuppMat.pdf.

## Data Availability

The data that support the findings of this study are available from the corresponding author upon reasonable request.
