# Supplementary material for: Maternal Vitamin D Deficiency is Associated With Sex‐Dependent Alterations in Neurodevelopment‐Related Gene Expression, Particularly in the Hypothalamus
Source: Mol Nutr Food Res. 2026 Oct 25;70(20):e70633. doi: 10.1002/mnfr.70633 (PMC13696975; doi:10.1002/mnfr.70633)
Supplement: Supplementary file 1 [file MNFR-70-e70633-s001.pdf]

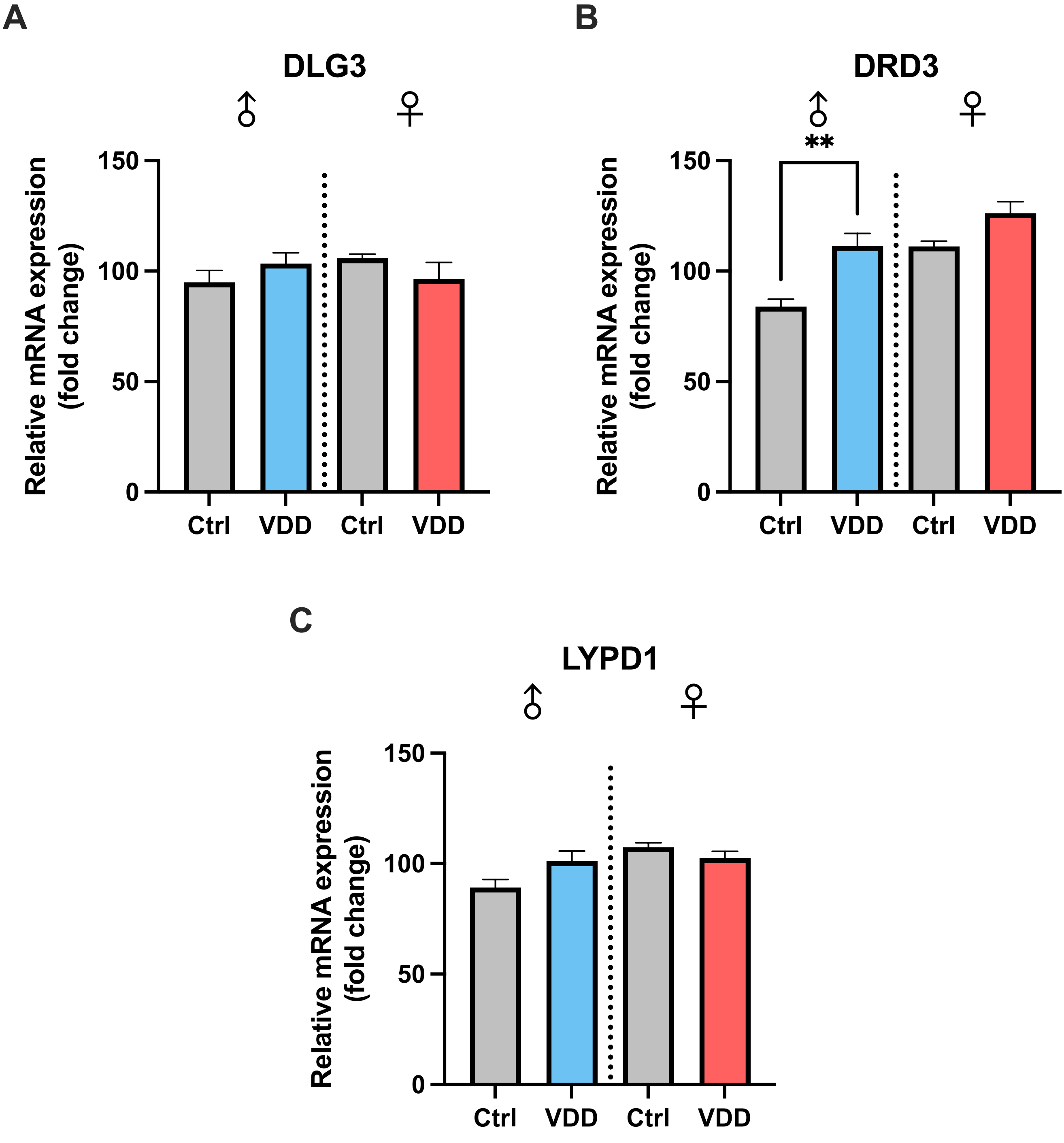

A
DLG3
♂
♀
150
100
50
0
Relative mRNA expression (fold change)
Ctrl
VDD
Ctrl
VDD
B
DRD3
♂
♀
**
150
100
50
0
Relative mRNA expression (fold change)
Ctrl
VDD
Ctrl
VDD
C
LYPD1
♂
♀
150
100
50
0
Relative mRNA expression (fold change)
Ctrl
VDD
Ctrl
VDD

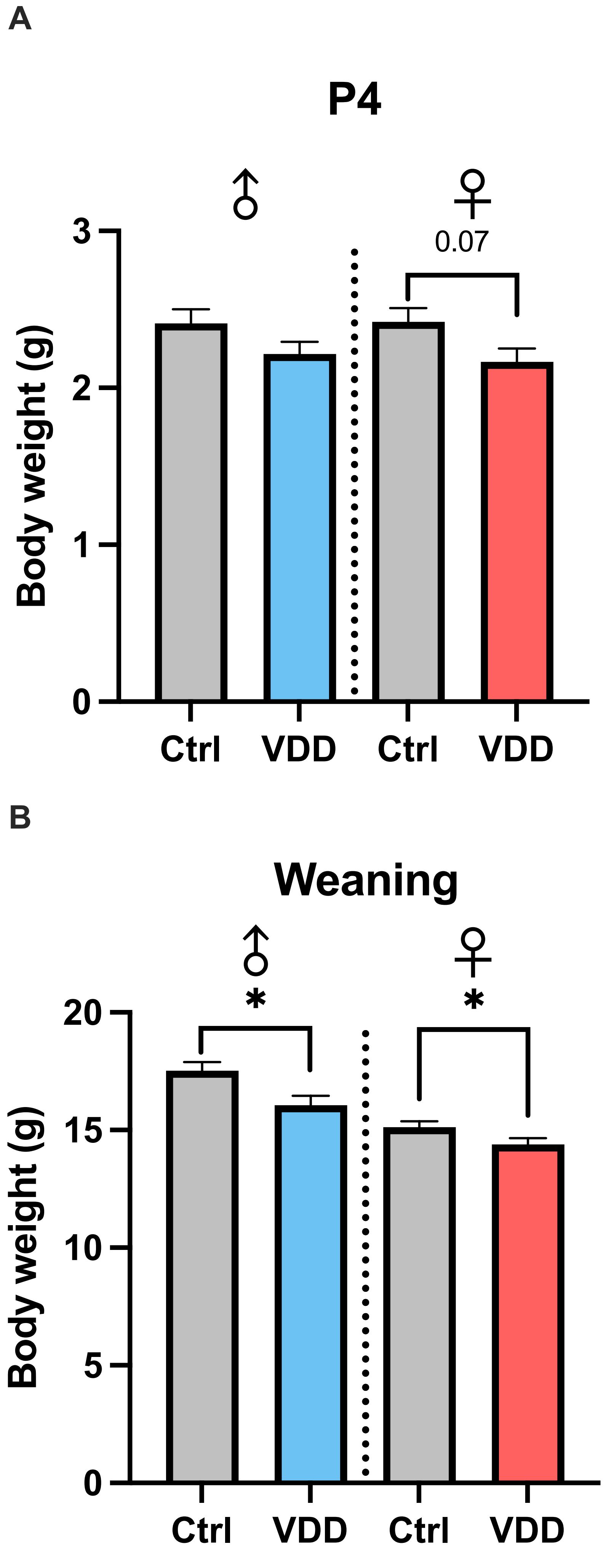
A
P4
♂
♀
0.07
Body weight (g)
3
2
1
0
Ctrl
VDD
Ctrl
VDD
B
Weaning
♂
♀
*
*
Body weight (g)
20
15
10
5
0
Ctrl
VDD
Ctrl
VDD

Figure S1. Maternal vitamin D deficiency altered hippocampus development gene expression in male offsprings at P4

Effect of maternal VDD on the levels of Disks Large Homolog 3 (*DLG3*) (A), Dopamine Receptor D3 (*DRD3*) (B), and LY6/PLAUR Domain Containing 1 (*LYPD1*) (C) in male and female offspring at P4. Ribosomal protein 18S rRNA was used as the endogenous control. Data were compared with Mann-Whitney (**$p < 0.01$) and are presented as mean ± standard error of the mean (SEM). N=10 ctrl male; 6 VDD male; 13 ctrl female, 5 VDD female Ctrl: control; VDD: vitamin D deficiency.

Figure S2. Body weight in maternal vitamin D deficiency offsprings at P4 and weaning.

Effect of maternal VDD on the body weight of male and female offsprings at P4 (A) and weaning (B). Data were compared with unpaired t test (*$p < 0.05$) and are presented as mean ± standard error of the mean (SEM). At P4, n=19 ctrl male; 12 VDD male; 28 ctrl female, 14 VDD female. At weaning, n=16 ctrl male; 15 VDD male; 13 ctrl female; 16 VDD female. Ctrl: control; g: grams; VDD: vitamin D deficiency.

Table 1. Sequence of the primers used for qPCR.

Gene Symbol	primer sequence Forward	primer sequence Reverse
18S	gtgggcctgcggcttaat	gccagagtctcgttcgttatc
AgRP	agctttggcggaggtgct	gcgacgcggagaacga
BDNF	gcatctgttggggagacaag	tcacctggtggaacattgtg
DLG3	aaccgggactggtatgagc	tgccattcaccgataggc
DRD3	accctggatgtcatgatgtg	ggcatgaccactgctgtgta
GFAP	acagactttctccaacctccag	ccttctgacacggatttggt
LYPD1	agtgatggagcaaagtgctg	tggtacccagctgaagcaat
NeuroD1	cgcagaaggcaaggtgtc	tttggtcatgtttccacttcc
NeuN	acctacagcatcggaaccat	ttgctagtagggggtgaagc
Npy	tccgctctgcgacactacat	tgctttccttcattaagaggtctg
Pomc	acgtggaagatgccgagatt	ggttttcagtcaggggctgt
PSD95	tctgtgcgagaggtagcaga	cggatgaagatggcgatag
Rbm31X/Y	caccttaagaacaagccaataca	ggcttgtcctgaaaacatttgg
SF1	cgtaaactgcgcacagga	tgtagattggttcaggggaag
Sim1	actcggctctcatctactcca	tgaaatgtacatgatcttcccatc
Syn-1	ggacggaagggatcacatta	tgagctgcttgtcttcatcc
Syp	caaggctacggccaacag	gtcttcgtgggcttcactg
TH	cccaagggcttcagaagag	gggcatcctcgatgagact
